# microRNAs, the Link Between Dengue Virus and the Host Genome

**DOI:** 10.3389/fmicb.2021.714409

**Published:** 2021-08-11

**Authors:** Yinghua Su, Ting Lin, Chun Liu, Cui Cheng, Xiao Han, Xiwen Jiang

**Affiliations:** ^1^College of Biological Science and Engineering, Fuzhou University, Fujian, China; ^2^DAAN Gene Co., Ltd. of Sun Yat-sen University, Guangdong, China

**Keywords:** microRNA, dengue virus, host–pathogen interaction, virus replication, signaling/signaling pathways

## Abstract

Dengue virus (DENV) is a small envelope virus of Flaviviridae that is mainly transmitted by *Aedes aegypti* and *Aedes albopictus*. It can cause dengue fever with mild clinical symptoms or even life-threatening dengue hemorrhagic fever (DHF) and dengue shock syndrome (DSS). At present, there are no specific drugs or mature vaccine products to treat DENV. microRNAs (miRNAs) are a class of important non-coding small molecular RNAs that regulate gene expression at the post-transcriptional level. It is involved in and regulates a series of important life processes, such as growth and development, cell differentiation, cell apoptosis, anti-virus, and anti-tumor. miRNAs also play important roles in interactions between host and viral genome transcriptomes. Host miRNAs can directly target the genome of the virus or regulate host factors to promote or inhibit virus replication. Understanding the expression and function of miRNAs during infection with DENV and the related signal molecules of the miRNA-mediated regulatory network will provide new insights for the development of miRNA-based therapies.

## Introduction

Dengue virus (DENV) is an important infectious agent of flavivirus, which is mainly transmitted by vector insects such as *Aedes aegypti* and *Aedes albopictus*. It can cause severe diseases in humans, such as dengue fever, dengue hemorrhagic fever (DHF), and dengue shock syndrome (DSS). DENV, an infectious virus prevalent in tropical and subtropical areas (Lambrechts et al., 2010) is widely distributed among humans, has a high incidence, and causes great harm. microRNAs (miRNAs), a small noncoding RNA (18–25 nucleotides) that exists widely in all kinds of plants, animals, and microorganisms, are a key transcription factor that affects gene expression. miRNAs can combine with 3'-untranslated regions (3'-UTRs) of messenger RNA (mRNA) to prevent translation events by degrading mRNA or inhibiting translation (Bartel, 2004), which may play an important role in the direct or indirect regulation of viral and host genome transcriptomes. At present, research on the interaction mechanism of miRNAs between DENV and host genome transcriptomes is lacking. Understanding miRNA expression and related target signaling pathways during DENV infection will provide new ideas for miRNA-based therapies. This paper reviews the regulatory role and mechanism of miRNA in DENV infection.

### Introduction to DENV

Dengue virus is a single-stranded positive-strand RNA (+ssRNA) virus that belongs to *Flaviviridae*. DENV can be divided into four serotypes according to its antigenicity: 1, 2, 3, and 4. The DENV genome is about 11 kb in length and 45–55 nm in diameter. The 5' and 3' ends of the virus RNA have untranslated regions containing several 100 nucleotides. The remaining DENV fragment is a single open reading frame. After infecting host cells, the viral genome is transcribed and translated into a total protein, which is cleaved into three structural proteins (C protein, prM/M protein, and E protein) and seven nonstructural proteins (NS1, NS2A, NS2B, NS3, NS4A, NS4B, and NS5; Perera and Kuhn, 2008) by viral and cellular proteases. Among them, E protein is the largest structural protein and the main envelope protein of DENV particles. Nonstructural proteins have many functions: They provide enzyme activity for biochemical reactions, and they provide suitable internal environments for viral RNA replication, including remodeling of the cell membrane and the inhibition of host antiviral reaction. Nonstructural protein 1 (NS1) has multiple polymer structures. In the early stages of infection, NS1 binds to the endoplasmic reticulum (ER) in the form of a dimer and anchors the viral replication complex (RCS) to the membrane. As the infection subsides, NS1 is secreted out of the cell in the form of a hexamer, which results in the body’s immune response to the virus (Muller and Young, 2013). NS2A, NS2B, NS4A, and NS4B are membrane binding proteins and components of the viral genome RCS. Among the four serotypes of DENV, NS5 has more than 70% homology and is the most conservative protein. NS5 has a variety of enzyme functions, including roles in N-terminal methyltransferase activity and C-terminal RNA-dependent RNA polymerase activity (Koonin, 1991; Klema et al., 2016). NS3 protein, a hydrophilic multifunctional protein with protease, RNA helicase, and RNA polymerase activity, plays an important role in processes, such as viral genome replication, packaging, and maturation (Tian et al., 2013).

## Pathogenesis of DENV and Host Immunity

### Invasion and Replication of DENV

Dengue virus infection begins with the bite of *A. aegypti* or *A. albopictus* infected by DENV, which can infect many kinds of cells, such as dendritic cells, macrophages, endothelial cells, and hepatocytes (Fang et al., 2013). The virus enters host cells through receptor-mediated endocytosis, in which the envelope glycoprotein E protein binds to receptors on the surfaces of host cells. Under neutral and slightly alkaline conditions, the E protein on the surface of the mature virus is in the form of a homodimer (Modis et al., 2004). In the acidic environment of the nucleosome, the E protein is restructured from a dimer to a trimer (Modis et al., 2004). This conformational change provides the energy required to bind the viral envelope to the host cell membrane and fuse the viral lipid bilayer and the cell membrane (Modis et al., 2004). E protein interacts with proteins on the surfaces of various mammalian and mosquito host cells during the invasion of the virus, but the real receptor for the virus has not yet been determined (Perera-Lecoin et al., 2013). The main receptors studied to date include heparin sulfate (Chen et al., 1997), dendritic cell-specific intracellular adhesion molecule-3-grabbing non-integrin (DC-SIGN) on dendritic cells (Tassaneetrithep et al., 2003), and mannose receptor (Miller et al., 2008). The virus enters the cell mainly through clathrin-mediated endocytosis (van der Schaar et al., 2008), which enables the viral genome to enter the cytoplasm. Subsequently, it uses the organelles and nutrients of host cells to synthesize genes and various proteins. The newly synthesized genome combines with capsids to form a nucleoprotein complex that sprouts into the ER cavity to infect the lipid bilayer (Yu et al., 2008). At the same time, the virus E protein and prM protein were produced (Yu et al., 2008). Immature virus particles are transmitted through the secretory pathway. In the Golgi network, prM protein lysis mediated by furin results in the rearrangement of E protein, homodimerization, and the formation of mature virus particles that are then released (Yu et al., 2008).

### Immune Regulation of Host Resistance to DENV

Once the virus invades cells and replicates, host cells can quickly recognize pathogen-associated molecular patterns through pattern recognition receptors (PRRs). For example, viral RNA, replication intermediates formed by virus nonstructural protease NS5, double-stranded RNA, and various proteins synthesized by the virus can initiate innate immune responses and guide adaptive immune responses to resist invasion by pathogenic microorganisms. PRRs include toll-like receptors (TLRs; Tsai et al., 2009), retinoic acid inducible gene I protein/melanoma differentiation factor 5(RIG-I/MDA5; Nasirudeen et al., 2011), and miRNAs (Trobaugh et al., 2014). The ability of these innate components to recognize DENV may have different effects on the life cycle of the virus and the host’s response to infection. In fact, the mutual recognition pattern of PAMP-PRR activates interferon regulatory factors, including IRF3, IRF7, and NF-κB. Interferon type I (IFN), chemokines, and cytokines are expressed to promote the expression of antiviral genes and the inflammatory response. When the virus infects the body, it can proliferates in the capillary endothelial cells and releases into the blood to form viremia, and it then further infects mononuclear macrophages in the blood and tissue to cause dengue fever. The occurrence of severe DHF and DSS may be mainly due to the effects of antibody-dependent enhancement (ADE; Halstead, 1982), cross-reactive T cell response (Mongkolsapaya et al., 2003), and cytokine storm (Clark, 2007).

### microRNA

RNA interference (RNAi) is a post-transcriptional gene-silencing mechanism mediated by small RNAs with length of 20–30 nucleotides, including miRNAs, small interfering RNAs (siRNAs), and PIWI-associated RNAs (piRNAs; Malone and Hannon, 2009; Castel and Martienssen, 2013). They can guide sequence-specific gene silencing in complex with an Argonaute protein (AGO protein) and cofactors and then involves the degradation of mRNA molecules, thereby preventing gene expression. It has demonstrated that RNAi phenomenon plays essential roles across eukaryotic organisms in genome defense against viruses and transposons: when the siRNAs are derived from viruses, they function as guides to specifically target the invading viruses for RNAi and thereby inhibit viral replication and infection (Castel and Martienssen, 2013). However, a role for cellular miRNAs in the defense against viral infection in mammalian organisms has thus far remained elusive (Cullen, 2006). Delightfully, RNA-based therapeutics, such as siRNAs, miRNAs, antisense oligonucleotides (ASOs), aptamers, synthetic mRNAs, and CRISPR–Cas9, have great potential to target a large part of the currently undruggable genes and gene products and to generate entirely new therapeutic paradigms in disease (Dowdy, 2017). A classical case is a liver-specific miRNA- miR-122, which is expressed almost exclusively in liver cells with more than 50,000 copies per cell (Filipowicz and Grosshans, 2011). It has been revealed that miR-122 is an essential host factor for hepatitis C virus (HCV) infection and an antiviral target, and clinical proof-of-concept studies have demonstrated that an miR-122 inhibitors Miravirsen, an LNA-modified anti-miR-122, can efficiently reduce viral load in chronically infected HCV patients without detectable resistance (Janssen et al., 2013; Bandiera et al., 2015).

### Biosynthesis of miRNA

In animals, miRNA genes are transcribed into primary transcripts (pri-miRNAs) by RNA polymerase II (RNA Pol II) in the nucleus, with a length of about 300–1,000 bases (Lee et al., 2004). In the nucleus exists a nuclear protein complex called a microprocessor complex that contains ribonuclease III endonuclease Drosha; DiGeorge syndrome chromosomal region 8 (DGCR8); and some minor cofactors, such as DEAD box RNA helicase P68 (DDX5) and p72 (DDX17). This microprocessor complex processes pre-miRNAs into hairpin structures of 70–90 nucleotides (Lee et al., 2003), namely, miRNA precursors. Subsequently, pre-miRNAs are transported from the nucleus to the cytoplasm by exportin5 protein (Bohnsack et al., 2004) and cleaved to form a Mir/Mir * complex of about 22 nt by Dicer1, an RNase III (Knight and Bass, 2001). Finally, one chain of the Mir/Mir * complex combines with ago (ago1 and ago2) protein to form the RNA-induced silencing complex (RISC). The chain that binds to the protein is called miRNA, and the other chain is called miRNA*. Mature miRNA is retained in the RISC, and miR * is released to and degraded in the cytoplasm (Krol et al., 2010). miRNA-bound RISCs mediate posttranscriptional silencing through two different mechanisms depending on their complementarity with target mRNA sequences (Bartel, 2004). Because of the endonuclease activity of ago2, complete complementary matching of miRNA and the target sequence usually leads to the degradation of mRNA, whereas an incomplete complementary combination of miRNA and the target sequence inhibits translation (Bartel, 2004).

### Regulation of miRNA

microRNA is an important factor regulating gene expression and is widely involved in physiological and pathological processes, such as early development, cell proliferation, apoptosis, cell death, the metabolism of fat, and so on (Bushati and Cohen, 2007). Each miRNA can have multiple target genes, and several miRNAs can regulate the same gene. This complex regulatory network can not only regulate the expression of multiple genes through one miRNA but also regulate the expression of a single gene through a combination of several miRNAs (Catalanotto et al., 2016). Mammalian miRNAs may control the activity of about 30% of protein coding genes and participate in the regulation of most cells; they are also closely related to most diseases (Filipowicz et al., 2008). Studies have shown the effects of miRNA deletion (Giraldez et al., 2005) and the role of miRNAs in signal transduction pathways (Boehm and Slack, 2005; Hu et al., 2020), cell proliferation (Brennecke et al., 2003), cell differentiation and apoptosis (Yin et al., 2020), and lipid metabolism (Xu et al., 2003). miRNAs also play an important role in infection with viruses. Viruses can use miRNAs to evade host immune monitoring and regulate host factors to promote their own replication. In contrast, miRNAs in host cells can promote or inhibit viral replication by targeting viral genome RNA and regulating their own host factors. A microarray study that used the blood of patients with acute DENV infection showed that the expression of 348 miRNAs changed after DENV infection; 17 miRNAs were found that may be used to distinguish mild dengue fever from severe DHF with complications (Tambyah et al., 2016). In another study, expression of broad-spectrum miRNAs in serum samples of three patients with DENV type 1 (DENV-1) and three healthy volunteers was analyzed with miRNA PCR array technology. A total of 41 miRNAs were upregulated and 12 miRNAs were downregulated in the serum of DENV-1 patients compared to healthy controls (Ouyang et al., 2016). Analyses of receiver operating characteristic (ROC) curves showed that serum hsa-miR-21-5p and hsa-miR-146a-5p can distinguish patients with dengue fever infection with good sensitivity and specificity. Moreover, functional analyses of these miRNAs show that they are involved in inflammation and cell proliferation. Therefore, these miRNAs are expected to be potential biomarkers for a diagnosis of DENV (Wen et al., 2015; Ouyang et al., 2016). Increasing numbers of studies have shown that miRNA plays an important role in DENV, which is discussed in detail below.

## The Role of Mirna in Regulating DENV and Related Signaling Pathways

### miRNAs Target the DENV Genome Directly to Inhibit or Promote DENV Replication

#### miRNAs That Inhibit DENV Replication by Targeting Viral Genomes

microRNAs usually induce translation inhibition by binding to MRE sites of target mRNAs. To date, many miRNAs have been identified that can affect DENV replication by directly targeting viral genome sequences. For example, miR-548 g-3p can regulate the replication of DENV-1, -2, -3, and -4 by directly targeting the stem ring structure of the virus 5'-UTR promoter element and can also interfere with the translation of DENV, thus inhibiting expression of the virus protein (Giraldez et al., 2005). Previous studies have found that the 5'-UTR of the DENV genome contains two defined elements essential for viral replication. At the 5' end, a large stem-loop (SLA) structure functions as the promoter for viral polymerase activity. Next to the SLA, there is a short stem-loop that contains a cyclization sequence known as the 5' upstream AUG region (5’UAR). The cis-acting elements in the 5'-UTR may involve in controlling viral protein translation, RNA synthesis, and encapsidation (Lodeiro et al., 2009; Gebhard et al., 2011). At the same time, overexpression of miR-484 and miR-744 can inhibit virus replication by acting on 3'-UTRs of the four serotypes of DENV, which indicates that miR-484 and miR-744 are two possible host factors inhibiting DENV infection (Castrillón-Betancur and Urcuqui-Inchima, 2017). Similarly, the 3'-UTR of the DENV is indispensable for their replication as they can promote the translation of the virus (Gebhard et al., 2011; Manzano et al., 2011). Castillo et al. (2016) found through bioinformatics analysis that host miR-133a can target 3'-UTRs of all four DENV serotypes and 3'-UTR of DENV downregulates endogenous expression of miRNA-133a in Vero cells during the first hours of infection. In addition, miR-133a overexpression inhibited DENV replication and this antiviral effect may be mediated by regulation of the host factor polypyrimidine tract binding (PTB) protein, a target of miR-133a (Castillo et al., 2016).

Yan et al. (2014) showed that miR-252 was highly expressed (more than three times uninfected) in a DENV-2 infection model of the mosquito C6/36 cell line and could downregulate expression of E protein through E protein gene targeting DENV-2, thus inhibiting DENV replication. It is noteworthy that the E protein of DENV plays an essential role in the process of virus attachment, fusion with host cell membrane and virus assembly, and can induce a protective immune response by neutralizing antibodies (Yu et al., 2008; Anasir et al., 2020). Therefore, understanding the impact of miRNA on DENV E protein has led to the exploration of miRNA-based drug discovery of antiviral. Finally, Lee et al. (2017) found that the water extract of Flos Lonicerae can upregulate expression of Let-7a in human and mouse blood, and Let-7a in turn can target the NS1 region of DENV-2 (nt 3,313–3,330) to inhibit the replication of DENV-2. Recent studies have revealed that DENV NS1 directly promotes vascular permeability by inducing a strong proinflammatory vasoactive response by mediating TLR 4 signaling (Stacey et al., 2015) and endothelial glycocalyx disruption (Puerta-Guardo et al., 2016). And it can activate platelets *via* TLR 4, leading to thrombocytopenia and hemorrhage (Chao et al., 2019). Therefore, this study provides a new insight for prevention and treatment of DENV infection through induction of the innate miRNA Let-7a by honeysuckle (Lee et al., 2017).

#### miRNAs That Promote DENV Replication by Targeting Viral Genomes

However, limited miRNAs can directly target viral genomes to promote viral replication but the specific mechanism remains to be investigated. Researchers screened a miRNA that was significantly differentially expressed after DENV-2 infection in HepG2 cells, namely, miR-21, and found that expression of miR-21 increased significantly and promoted the replication of DENV-2 after viral infection of HepG2 cells. However, prior to DENV infection of HepG2 cells, a significant reduction in DENV-2 production was observed after treatment with the miR-21 antagonist anti-miR-21 (AMO-21); thus, miR-21 is expected to be an intervention target for dengue treatment (Kanokudom et al., 2017). However, the mechanism of the replication of DENV induced by miR-21 is unclear; miR-21 may directly target the NS1 protein sequence of the DENV-2 genome (Miranda et al., 2006). Besides, Zhou et al. (2014) found a midgut-specific miRNA in an *A. albopictus* vector, namely, miR-281, after mosquito C6/36 cells were infected with DENV-2. miR-281 may target the 5'-UTR of DENV-2 genomic RNA and upregulate in response to viral infection, thus promoting DENV replication, whereas antagonism or knockout of miR-281 reduces the viral RNA level in *A. albopictus* (Zhou et al., 2014). These miRNAs, which inhibit or promote viral replication by targeting viral genomes, play a direct role in regulating viral infection and reasonable regulation of miRNAs expression and may avoid the excessive production of cellular inflammatory factors, thus avoiding the risk of DHF. miRNA-based therapeutics, such as miRNA mimics and inhibitors of miRNAs may have great potential to directly regulate virus replication and thus treat DENV-related diseases.

**Figure 1:**
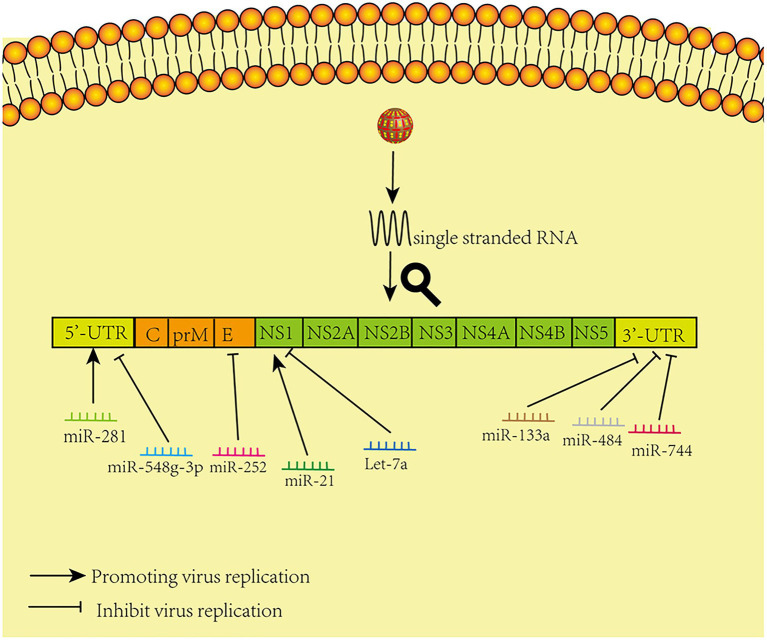
Model of miRNAs that directly target viral genomes. The figure shows that different microRNAs promote and inhibit virus replication by acting directly on corresponding sites in the virus genome.

Relevant summaries of miRNA are shown in Table 1 and in the schematic diagram in Figure 1.

**Table 1:** microRNAs that directly target viral genomes to inhibit or promote viral replication.

miRNA	Experimental model	Target	Effects	References
miR-548 g-3p	U937	DENV 5’-UTR	Inhibit	Wen et al., 2015
miR-484, miR-744	Vero	DENV 3’-UTR	Inhibit	Castrillón-Betancur and Urcuqui-Inchima, 2017
miR-133a	Vero	DENV 3’-UTR	Inhibit	Castillo et al., 2016
miR-252	C6/36	Viral gene E	Inhibit	Yan et al., 2014
Let-7a	Blood of humans and mice	NS1 sequence (nt:3,313–3,330)	Inhibit	Lee et al., 2017
miR-21	HepG2	NS1 sequence	Promote	Miranda et al., 2006; Kanokudom et al., 2017
miR-281	C6/36	DENV 5’-UTR	Promote	Zhou et al., 2014

### miRNAs Inhibit or Promote DENV Replication by Regulating Host Factors

Since viruses are parasitic organisms that rely on a range of host cytokines for replication and infection, many miRNAs have been shown to indirectly regulate DENV replication by regulating host factors or immune responses. miRNAs can affect the process of viral translation and replication by acting on related cytokines and regulating related signaling pathways. A large number of studies have clarified how related miRNAs affect related signaling pathways and thus regulate viral replication and disease progression (Barbu et al., 2020). Besides, miRNAs can also enhance or limit cellular responses to infection, such as immune responses or defense mechanisms (Trobaugh et al., 2014).

#### miRNAs That Inhibit DENV Replication by Regulating Host Factors

##### miRNAs That Inhibit DENV Replication by Regulating IFN System

After the virus invasion, the body will take the lead in initiating the innate immune response. And the type I interferon system inhibits viral infection by establishing a homeostasis in both infected and uninfected cells. DENV infection triggers the production of type I IFN after viral RNA is detected by TLRs and RIG-I-like (RLR) receptors, and signaling cascades are initiated *via* connector molecules, such as MyD88, MAVs, or STING (Diamond and Pierson, 2015). Type I IFN binding to cells induces a JAK-STAT-dependent signaling cascade that ultimately leads to the expression of hundreds of IFN-stimulating genes (ISGs), some of which block specific steps in the DENV lifecycle (Schoggins et al., 2012). However, DENV is able to resist the production of type I IFN and signal transduction of key myeloid targets. In addition to regulating DENV replication, the antagonistic type I IFN signaling pathway in antigen-presenting cells attenuated or skewed the adaptive immune response (Diamond and Pierson, 2015).

**Figure 2:**
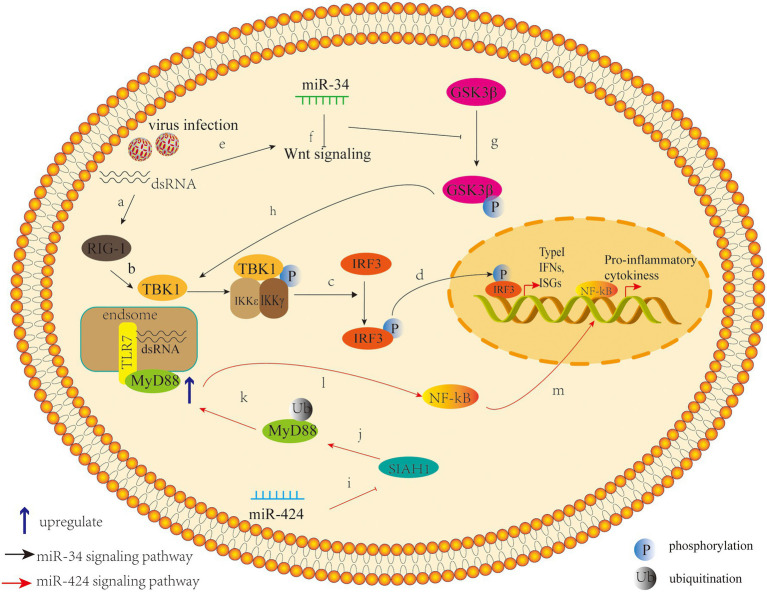
Pattern of miR-34 (the black arrow) and miR-424 (the red arrow) that inhibit or promote DENV replication by regulating host factors. (a) The induction of the innate response by virus infection or other stimuli through pattern recognition/retinoic acid inducible gene I (RIG-I)-like receptors. (b)Viral infection leads to activation of innate immunity and first induces phosphorylation of TANK-binding kinase 1 (TBK1). (c) This is followed by the phosphorylation or homodimerization of the interferon regulatory factor 3 (IRF3), (d) and then it translocated into the nucleus, where it induces the transcription of type I Interferons (IFNs) and interferon-stimulated genes. (e) Meanwhile, the viral infection also activate the Wnt signaling pathway, (f) leading to the repression of the Glycogen synthase kinase 3 beta (GS3Kβ) phosphorylation, and (g) which suppresses the IFN pathway by interacting with TBK1. The intersection between these two pathways suggests that the Wnt signaling pathway has the ability to modulate the innate inflammatory response. (h) miR-34 can act as an inhibitor of the Wnt signaling pathway to enhance type I IFN signaling, leading to a cellular antiviral status (Smith et al., 2017). (i) miR-424 suppresses expression of the E3 ubiquitin ligase SIAH1; (j) SIAH1 binds to and ubiquitinates the innate immune adaptor protein MyD88; (k) SIAH1 knockdown inhibits proteasome-dependent degradation of MyD88; (l) Inhibition of SIAH1 increases MyD88-Mediated NF-kB signaling during DENV2 infection; (m) and further promote the production of the NF-kB–activated proinflammatory cytokines.

A previous study has demonstrated that mammals can use cellular miRNAs to fight viral infections through the interferon system (Pedersen et al., 2007), and several studies have confirmed that dysregulated miRNAs can mediate the interferon system to regulate viral replication after DENV infection. For example, expression of Let-7c is upregulated and it has been shown to have a positive effect on protecting cells from oxidative stress and inflammation when DENV infects the human hepatoma cell line Huh-7 and monocyte macrophage line U937-DC-SIGN, and Let-7c directly targets the Basic Leucine Zipper Transcription Factor-1 (BACH1; Escalera-Cueto et al., 2015). However, BACH1 inhibited the expression of heme oxygenase-1 (HO-1) and antioxidant genes involved in oxidative stress response. Therefore, the upregulation of Let-7c expression indirectly inhibits the replication of DENV-2 and DENV-4 in Huh-7 cells through the modulation of host factors like BACH1 and HO-1. The research of miRNAs that modulate their expression after DENV infection in other cell types may give useful clues about the host molecules that may participate during viral infection (Escalera-Cueto et al., 2015). Another study showed that the antiviral effects of HO-1 are related to the recovery of the antiviral IFN immune response signal pathway and the inhibition of DENV protease activity by biliverdin, a byproduct of heme degradation catalyzed by HO-1 (Tseng et al., 2016). A recent study found that overexpression of miR-155 in Huh-7 cells inhibits virus replication *in vitro* (Su et al., 2020). *In vivo*, overexpression of miR-155 can protect ICR suckling mice from the threat of DENV infection, and miR-155 also induces HO-1-mediated antiviral response by targeting BACH1 (Su et al., 2020). Finally, the HO-1-mediated inhibition of NS2B/NS3 protease activity enhances induction of antiviral interferons and inhibits virus replication (Su et al., 2020). These studies suggest that HO-1 and related miRNAs can be used as potential therapeutic targets to control DENV replication. Related studies have shown that miR-155 regulates immune cells, including dendritic cells, B cells, and T cells, and targets essential molecules involved in regulating the immune system as well as participating in multiple signaling pathways, including MAPK, insulin, Wnt, and MAPK/NF-κB (Pashangzadeh et al., 2021). In view of this, miR-155 may also play a crucial role in the immune regulation of DENV by targeting a wide range of pathways across different immune responses. Zhu et al. (2014) found that expression of miR-30e* is upregulated in HeLa cells and U937 cells infected by DENV and then activates the NF-κB pathway by targeting IKBα, an inhibitor of the innate immune signaling pathway of the virus 3'-UTR. Thus, it promotes expression of IFN-β and its downstream genes, such as oligoadenylate synthase-1 gene (OAS1), myxovirus resistance protein A gene (MxA), and interferon-induced transmembrane protein 1 gene (IFITM1), thus inhibiting virus replication (Zhu et al., 2014). In a previous study on cancer, microRNA-30e * was also reported to promote human glioma cell invasion in an orthotropic xenograft model by directly targeting the IκBα 3'-UTR and suppresses IκBα expression and then disrupting the NF-κB/IκBα negative feedback loop (Jiang et al., 2012). This miRNA-mediated epigenetic regulation may lead to a more efficient and direct activation of NF-κB in a cascading manner than the phosphorylation and ubiquitination mediated activation by IKK-induced phosphorylation. Hence, these studies emphasize the direct regulation of miR-30e* on NF-kB signaling pathway, which is an important intracellular nuclear transcription factor involved in the body’s inflammatory response and immune response. Another study found that the miR-34 family (including miR-34a, miR-34c, miR-449a, and miR-449b) has similar natural regulatory effects in response to a variety of flavivirus infections, including DENV (Smith et al., 2017). Upregulation of miR-34 family expression inhibits the Wnt signaling pathway, resulting in the inhibition of phosphorylation of glycogen synthase kinase 3 (GSK3β). GSK3β sends positive feedback to the IFN signal pathway by interacting with serine kinase TBK1, which promotes phosphorylation of IRF3 and subsequent transcriptional activation of IFN-I and other ISGS, thus promoting antiviral response (Smith et al., 2017). The miR-34 family increases production of IFN-I and expression of ISGS by inhibiting the Wnt signaling pathway and thus inhibits virus replication (Smith et al., 2017; Figure 2). In fact, miR-34a has demonstrated various regulatory effects on cancer cells by targeting multiple genes and signaling pathways, and has shown great promise in animal models of myeloma, hepatocellular carcinoma, lung cancer, and breast cancer (Misso et al., 2014). It is currently being tested in human trials on the efficacy of hepatic and hematologic cancers (Misso et al., 2014). These studies highlight the role of miR-34a in the antiviral response and the possibility that small molecule antagonists in the Wnt signaling pathway that have been suggested as anticancer therapies (Dihlmann and von Knebel, 2005) may be important antiviral targets.

In short, these studies emphasize that miRNA mediates the regulation of interferon system in immune response by related target genes, and then triggers the host’s antiviral response.

##### miRNAs That Inhibit DENV Replication by Regulating Other Host Factors

The intracellular environment is complex and involves numerous signaling pathways, which are also crucial for cellular metabolism. Many miRNAs can also influence viral replication by regulating cytokines other than immune responses. Diosa-Toro and other scholars have found another miRNA with antiviral effects, namely, miR-3614-5p (Diosa-Toro et al., 2017). They found that when human macrophages were not infected with DENV, expression of miR-3614-5p was upregulated, and this overexpression reduced the infectivity of DENV (Diosa-Toro et al., 2017). The researchers predicted that adenosine deaminase acting on RNA1 (ADAR1), a protein that can promote viral replication, is one of the targets of miR-3614-5p and proved that ADAR1 can improve the infectivity of DENV in the early stages of cell infection with the virus (Diosa-Toro et al., 2017). This study extends the knowledge of the contribution of human miRNAs in the construction of networks of interactions between DENV and its human host cells. Wu et al. (2014) showed that miR-223 was significantly decreased after infection with DENV-2 in human endothelial Eahy926 cells and that this overexpression inhibited the replication of DENV-2. This is because miR-223 downregulates the expression of STMN1 through the STMN1 gene, a microtubule instability protein targeting the 3'-UTR of viral RNA, thus inhibiting virus replication (Wu et al., 2014). STMN1 is a microtubule depolymerization protein that is closely related to the formation of spindle bodies in cell mitosis. Therefore, STMN1 can regulate mitosis and may affect the life cycle of DENV. However, the exact mechanism of how STMN1 affects the replication of DENV-2 remains to be elucidated.

Ritu Mishra and other scholars have explored the role of miRNA in mediating the regulation of deubiquitinases in the host proteasome (Mishra et al., 2019). Ubiquitin-specific proteinase 42 (USP42) is a deubiquitination enzyme that is widely expressed in various human tissues (Quesada et al., 2004). USP42 is co-localized with RNA Pol II in the nucleus, binds with histone H2B, and deubiquitinates H2B. The decrease in USP42 expression regulates the ubiquitination of H2B and inhibits the basic and induced transcription of many promoters (Hock et al., 2014). DENV infection causes a dose-dependent downregulation of host DUB-USP42, and the DENV-NS5 gene alone is enough to cause this downregulation (Mishra et al., 2019). When NS5 is overexpressed, miR-590 is upregulated in a dose-dependent manner. Luciferase assay confirmed the direct regulatory interaction between miR-590 and the 3'-UTR of USP42 (Mishra et al., 2019). This study showed that DENV infection upregulates expression of miR-590 in human microglia, then downregulates expression of host ubiquitinase protein-USP42, thus inhibiting the replication of DENV (Mishra et al., 2019). Besides, the regulation of miR- 590 by mimics and antagonists also affects the expression level of TRAF6, a major regulator of host inflammatory response (Mishra et al., 2019). This study provides a new insight for miR-590 to regulate virus replication by mediating host proteasome. Besides, miR-590 also showed dysregulation in the sera of dengue infected patients (Ouyang et al., 2016). These results hint us that miR-590 plays a crucial role in DENV infection, but its exact regulatory mechanism remains to be elucidated.

Another study found that miR-424 inhibits expression of E3 ubiquitin ligase SIAH1 by activating unfolded protein response (UPR) during the infection of HeLa cells by DENV-2 (Murphy Schafer et al., 2020). The downregulation of SIAH1 can inhibit the replication of DENV, which indicates that this target plays a role in the antiviral activity of miR-424, at least to a certain extent (Murphy Schafer et al., 2020). SIAH1 binds to MyD88, an important transducing protein in the TLR signaling pathway, and ubiquitinates it. Moreover, the antiviral effect of SIAH1 is reduced in cells from which MyD88 has been deleted by CRISPR/Cas9 gene editing (Murphy Schafer et al., 2020). In addition, in cells in which SIAH1 has been downregulated by miR-424 or siRNA knockout by SIAH1, the MyD88-mediated NF-kB signaling pathway is enhanced by DENV-2 infection or by imiquimod, a TLR7 ligand (Murphy Schafer et al., 2020; Figure 2). This study indicates that the target SIAH1 plays an important role in the antiviral activity of miR-424 to some extent and hints us that an additional pathway by which DENV2 harnesses aspects of the UPR to dampen the host innate immune response and promote viral replication.

#### miRNAs That Promote DENV Replication by Regulating Host Factors

As viruses continue to evolve, they have also gradually evolved a set of new mechanisms to evade host immune monitoring or use host factors to promote their own replication. After viral infection, some dysregulated miRNAs perform a role of promoting viral replication.

As we know, immune-mediated cytokine storms plays a vital role in understanding the pathogenesis of dengue fever (Srikiatkhachorn et al., 2017). DENV infection induces massive immune activation and the production of high amounts of proinflammatory cytokines, such as tumor necrosis factor-alpha (TNF-α) and IFN, which may contribute to the immunopathogenesis of severe DENV infection, such as DHF/DSS (Srikiatkhachorn et al., 2017). Wu et al. (2013) found that expression of miR-146a was significantly upregulated after DENV-2 infection of human primary monocytes and human peripheral blood monocytes and that miR-146a inhibits the production of IFN-β by targeting tumor necrosis factor receptor-associated factor 6 (TRAF6). Another study found that Enterovirus 71 upregulates expression of miR-146a through mediating activator protein 1 (Ho et al., 2014). Moreover, miR-146a targets interleukin-1 receptor-associated kinase (IRAK1) and TRAF6 to mediate the TLR signaling pathway and reduce the production of IFN-I, to escape the immune attack of the host (Ho et al., 2014). Knockout or neutralization of virus-induced miR-146a blocks this signaling cascade, restores IRAK1 and TRAF6 expression, increases IFN-I production, and thus inhibits viral replication and improves mouse survival (Ho et al., 2014). This study provides new insight into whether other viruses also evade host immune defense mechanisms through similar mechanisms. Pu et al. (2017) confirmed that miR-146a negatively regulates the autophagy process of A549 cells and THP-1 cells by targeting TRAF6; that is, overexpression of miR-146a significantly blocks DENV-2 induced autophagy, whereas the inhibition of miR-146a expression mediated by locked nuclear acid (LNA) counteracts this effect. Based on this finding, the miR-146a antagonist may play an essential role in restoring the production of host IFN and inhibiting viral replication.

And some cytotoxic molecules, such as perforin and granzymes may involve in cytokine storms (Mongkolsapaya et al., 2006; Mathew and Rothman, 2008). Liu et al. (2016) identified the regulatory role of miR-378 in a serine esterase granzyme B (Grzb), which can induce target cell death through multiple pathways, and the protective effects of Grzb in inhibiting DENV replication *in vivo*. Expression of miR-27a *, miR-30e, and miR-378 was downregulated in patients with DENV infection, and expression of Grzb in natural killer (NK) cells and CD8+ T cells was significantly increased by DENV infection (Liu et al., 2016). Further studies have shown that the main source of Grzb is NK cells. miR-378 rather than miR-27a * or miR-30e inhibits expression of Grzb in NK cells, and overexpression of miR-378 in DENV-infected mice can inhibit expression of Grzb and promote the replication of DENV (Liu et al., 2016). Previous studies have found that I type IFN stimulation enhanced the expression of cytolytic molecules by downregulating miRNA-378 and miR-30e, two kinds of miRNAs with abundant expression, and thus enhanced the cytotoxicity of human NK cells through analysis of miNome in human NK cells (Wang et al., 2012). Therefore, the possibility of miR-378 regulating viral replication by mediating the interferon system cannot be ruled out.

Finally, miR-927 can promote replication of the virus in C6/36 mosquito cells during acute and persistent infection with DENV-2 (Avila-Bonilla et al., 2020). A double luciferase gene reporter experiment confirmed that Filamin (FLN) is a direct target of miR-927 and that FLN is essential in actin rearrangement and related to the regulation of the toll pathway (Avila-Bonilla et al., 2020). Several studies have demonstrated that the Toll pathway plays an essential role as an anti-DENV mechanism in both *A. aegypti* mosquitoes (Xi et al., 2008; Luplertlop et al., 2011) and mosquito cells (Sim and Dimopoulos, 2010). Overexpression or downregulation of miR-927 leads to changes in Cecropin A, G, and Defensin D expression in toll pathway reactions (Avila-Bonilla et al., 2020). In conclusion, this study confirmed that miR-927 is an important factor regulating DENV-2 acute and persistent infection in mosquito cells, and may regulate innate immune response by inhibiting FLN to promote DENV infection, thereby performing a pro-viral activity (Avila-Bonilla et al., 2020). Relevant miRNAs are summarized in Table 2.

**Table 2:** microRNAs that inhibit or promote dengue virus (DENV) replication by regulating host factors.

miRNA	Experimental model	Target	Effects	References
Let-7c	Huh-7, U937-DC-SIGN	BACH1	Inhibit	Escalera-Cueto et al., 2015; Tseng et al., 2016
miR-155	Huh-7	BACH1	Inhibit	Su et al., 2020
miR-30e*	HeLa, U937, PBMCs	IκBα	Inhibit	Zhu et al., 2014
miR-34 family	HeLa	Wnt pathway	Inhibit	Smith et al., 2017
miR-3,614-5p	Primary human macrophage	ADAR1 mRNA	Inhibit	Diosa-Toro et al., 2017
miR-223	EAhy926 cells	STMN1 mRNA	Inhibit	Wu et al., 2014
miR-590	Human microglia cells	USP42	Inhibit	Mishra et al., 2019
miR-424	HeLa	SIAH1	Inhibit	Murphy Schafer et al., 2020
miR-146a	Primary human monocytes and THPI cells	TRAF6	Promote	Wu et al., 2013
miR-378	Human NK cells	Gzmb in NK cells	Promote	Liu et al., 2016
miR-927	C6/36	FLN	Promote	Avila-Bonilla et al., 2020

In conclusion, these findings highlight opportunities to use miRNAs as tools for discovering and describing the unique cytokines involved in promoting or limiting viral replication, which will open up new avenues for antiviral research. The feasibility of miRNA-based treatments for DENV is worthy of further study.

### miRNAs Can Be Used as Diagnostic Markers

Nowadays, a series of studies has revealed that miRNAs can be used as biomarkers in the diagnosis of some diseases. For example, miR-486-5p (Tian et al., 2019) and miR-200a-3p (Di et al., 2020) have been identified as potential diagnostic and prognostic markers for lung cancer and colorectal cancer, respectively. In addition, several miRNAs related to cardiac metabolism have been found that can participate in lipid metabolism and can be regarded as potential biomarkers for the early diagnosis or progress of type II diabetes and coronary heart disease (Mens et al., 2020). Dynamic changes in serum miR-122 and its content at weeks 12 and 24 can be used as independent predictors of virologic response in patients with chronic hepatitis B with high viral loads being treated with nucleoside analogues (Wu et al., 2019). Chen et al. (2014) revealed the relationship between miR-150 and suppressors of cytokine signaling (SOCS1) in DENV-2 infection of peripheral blood mononuclear cells (PBMCs). In this study, expression of SOCS1 and gamma interferon (IFN-γ), an antiviral, antitumor, and immunoregulatory agent, was significantly reduced in monocytes of DHF patients. Moreover, 24 h after infection of PBMCs with DENV-2, increased SOCS1 expression and decreased miR-150 expression were detected. When miR-150 simulators were transfected into CD14(+) cells infected with DENV-2, it inhibited the induction of SOCS1 expression in a dose-dependent manner (Chen et al., 2014). This study emphasized that the upregulation of miR-150 expression in CD14(+) cells is related to the downregulation of SOCS1 expression, and the pathogenesis of DHF and miR-150 may serve as a potential biomarker (Chen et al., 2014). Another study investigated some dysregulated miRNAs in 20 patients with dengue fever and 20 patients with severe dengue fever. It was found that the expression of miR-150 in peripheral blood cells of patients with severe dengue fever was significantly higher than that of patients with dengue fever, and miR-150 could target zeste homolog 2 enhancer (EZH2; Hapugaswatta et al., 2020), a factor which is involved in the induction of multiple inflammatory, stress, and antipathogen pathways (Arbuckle et al., 2017). Therefore, in the early stage of dengue infection, the differential expression of miR-150 and EZH2 may be serve as reliable biomarkers of disease severity. Furthermore, in a recent study, high-throughput sequencing of RNA from plasma samples of 39 dengue patients revealed that circulating microRNAs could distinguish different stages of dengue disease, so they have the potential to serve as a marker for dengue disease progression (Saini et al., 2020).

### Limitations of Research on DENV-Associated miRNAs

Taken together, we summarized the existing relationship between cellular miRNAs and viral infection, miRNAs can not only directly target the viral genome to regulate the life cycle of the virus, but also mediate host innate and adaptive immune processes by regulating various host factors, which may closely be related to the incidence and severity of dengue fever. Although growing evidence has demonstrated the association between cellular miRNAs and viral infection and some miRNAs involved in inflammatory response has been described, these studies have mainly focused on cell models and little is known about the relationship between miRNAs and the pathogenesis of DHF. A recent study sequenced miRNomes from six deaths and compared them with five controls to characterize microRNAs expression profiles in human liver tissue during DHF (de Oliveira et al., 2021). Eight microRNAs were found to be differentially expressed, including endothelial cell regulatory molecule miR-126-5p, liver specific homeostasis regulator miR-122-5p, and interferon regulator miR-146a-5p. Enrichment analysis with predicted target genes of microRNAs revealed regulatory pathways of apoptosis, involving MAPK, RAS, CDK, and FAS (de Oliveira et al., 2021). Immune response pathways were related to NF-kB, CC and CX families, IL, and TLR. This is the first description of the human microRNA and isomicroRNA profile in liver tissues from DHF cases and the results demonstrated the association of miR-126-5p, miR-122-5p, and miR-146a-5p with DHF liver pathogenesis, involving endothelial repair and vascular permeability regulation, control of homeostasis, and expression of inflammatory cytokines (de Oliveira et al., 2021). However, in clinical treatment, tissue samples are not readily available, and it is desirable to study the relationship between miRNA and the pathogenesis of DENV infection and DHF in serum or blood samples. Above, we referred to enhanced miR-150 expression is associated with depressed SOCS1 expression involved in DHF (Chen et al., 2014) and we have discussed how differential expression of miRNAs in blood samples in clinical patients can be used to distinguish between the severity of dengue patients (Tambyah et al., 2016), and dysregulated miRNAs in serum samples may mediate inflammatory responses and cell proliferation (Ouyang et al., 2016), which expands our understanding of the pathogenesis of miRNAs and DHF *in vivo* studies.

Existing research has shown that serum miRNAs have many innate advantages: they exist widely in eukaryotes and are highly conserved and it is only expressed in specific tissues and is tissue specific; miRNA has specific expression in different growth and development stages of cells, which has a time sequence. Besides, the expression of miRNA was inhibited at the level of protein translation, so the change of miRNA in disease was earlier than that of protein markers (Brase et al., 2010). The above characteristics make miRNAs have the potential to become a new disease detection marker. At present, the detection of serum miRNA is still in the early stage of research, and there are several problems as follows: the detection method is complicated, and the price is relatively expensive, which is not conducive to extensive clinical application. In addition, the detection of serum miRNAs needs to be standardized and the reference range of serum miRNA as disease-related markers needs further demonstration. Finally, the sample size of clinical trials is generally small, and there is a lack of long-term follow-up data (Brase et al., 2010). Furthermore, secreted miRNAs, especially those in Extracellular Vesicles (EVs) such as exosomes, may mediate paracrine and endocrine communication between different tissues and thus modulate gene expression and the function of distal cells (Mori et al., 2019).

## Summary

Several studies have shown that miRNA plays an important role in DENV infection. However, studies of the targets of miRNAs and the regulatory network associated with the host or viral genome transcriptome of miRNA are still limited, and descriptions of the relevant signaling pathways of miRNA in DENV infection, replication, and host immunity are lacking. At present, some researchers are trying to develop more scientific and accurate methods of predicting miRNA targets (Quillet et al., 2019) and to integrate multiple regulatory networks to predict the function of miRNA (Deng et al., 2019), but these studies are far from sufficient. Moreover, because of the lack of suitable animal models for studying DENV infection, the functions of many host factors still need to be evaluated *in vivo*. One biological characteristic of miRNA is that it can target multiple mRNAs, so the pathophysiological effects observed by regulating miRNA expression may be related to subtle changes in the mRNA levels of different targets (Nguyen et al., 2018). Meanwhile, each mRNA molecule may contain several or even dozens of potential miRNA targets, so the specificity of miRNA inhibition on target genes requires further study, and accurate means of evaluating the miRNA genome transcriptome should be developed. In addition, because miRNA may be the main factor regulating disease and inflammation, the improper design of miRNA operations may have unexpected side effects, so it is necessary to develop excellent vectors to precisely limit the release of miRNA mimics and inhibitors to infected and inflammatory tissue (Nguyen et al., 2018). Moreover, abnormal expression of miRNA may lead to innate immune and adaptive immune deregulation, resulting in failure to resist invasive pathogens and autoimmune diseases (Lee et al., 2014), which have negative impacts on host health. In conclusion, in-depth understanding of miRNAs and miRNA-mediated signaling pathways in DENV replication and pathology will provide insights for the development of targeted drugs.

## Author Contributions

YS, TL, and CL: literature search. YS, TL, CL, and CC: figures. XH and XJ: study design. YS: writing. All authors contributed to the article and approved the submitted version.

## Conflict of Interest

XJ is employed by DAAN Gene Co., Ltd. of Sun Yat-sen University.

The remaining authors declare that the research was conducted in the absence of any commercial or financial relationships that could be construed as a potential conflict of interest.

## Publisher’s Note

All claims expressed in this article are solely those of the authors and do not necessarily represent those of their affiliated organizations, or those of the publisher, the editors and the reviewers. Any product that may be evaluated in this article, or claim that may be made by its manufacturer, is not guaranteed or endorsed by the publisher.
